# Cannabis use and cannabis use disorders and their treatment in the Europe

**DOI:** 10.1007/s00406-024-01776-1

**Published:** 2024-03-15

**Authors:** Wayne Hall, Jakob Manthey, Daniel Stjepanović

**Affiliations:** 1 The National Centre for Youth Substance Use Research, The University of Queensland, St Lucia, Australia; 2 Institute of Clinical Psychology and Psychotherapy, Technical University Dresden, Dresden, Germany

**Keywords:** Cannabis use, Cannabis use disorders, Public education, Screening, Brief interventions, Specialist treatment

## Abstract

This paper introduces the special issue on cannabis use in Europe. It describes data on the prevalence of cannabis use in Europe and the more limited data on the prevalence of cannabis use disorders, one of the most common forms of drug problem treated in many countries in Europe. It summarises what research has indicated about the adverse effects of acute and chronic cannabis use and discusses potential health system responses that may reduce some of these harms. These include public education about the risks of cannabis use; screening and brief interventions in primary medical settings; and specialist treatment for cannabis use disorders. It briefly indicates the special issues that may need to be addressed in dealing with the high rates of comorbidity between cannabis use disorders, other types of drug use disorders, and common mental disorders.

Cannabis is one of the most widely used illicit drugs in the European Union [37] and in many other high-income countries [47, 59]. Cannabis use disorders are one of the most common types of drug problem treated in Europe [17]. We use the term “cannabis use disorders” to describe problems with cannabis use that lie on a continuum of severity that ranges from mild to moderate problems in controlling cannabis use through to more severe problems as indicated by meeting criteria for a diagnosis of cannabis dependence.

In the early 1990s in the USA the lifetime risk of a cannabis use disorder was estimated at 9%, increasing to 16% in persons who initiated use in adolescence [1]. A meta-analysis of studies conducted since then, suggests that the risk of developing cannabis use disorders may be one in five among persons who have ever used cannabis, and one in three among persons who have used cannabis at least weekly for an extended period [34].

## Cannabis use in the European Union

In 2023, EMCDDA estimated that 22.2 million adults aged 15–64 had used cannabis in the past year (8.0% of this age group). The prevalence of cannabis use was 15.3 million among Europeans aged 15–34 years (15.1%). The prevalence of use in the past year in the 15–34-year age group varied from 22.9% in Czechia to less than 1.8% in Turkey.

Recent use of cannabis increased between 2010 and 2019 in 24 of 26 countries providing data to EMCDDA [37]. The proportion of past-month cannabis users who were near daily users varied from less than 10% to 50–70% and was 20% or more in half of the EU member states [37]. An estimated 1.3% of adults aged 15–64 years were daily or almost daily cannabis users, and 57% were under the age of 35 [17]. An increase in the prevalence of daily and near daily cannabis use was most pronounced in Portugal and Spain.

The 2019 European School Survey Project on Alcohol and Other Drugs (ESPAD) study collected data from 16-year-old students in 24 European countries [15]. On average, 16% of students had used cannabis at least once and boys reported more lifetime cannabis use than girls (18% versus 13%). The countries with the highest prevalence were Czechia (28%), Italy (27%) and Latvia (26%). The lowest rates of cannabis use (2.9–7.3%) were in Kosovo, North Macedonia, Iceland and Serbia.

Overall, 7.1% of the students surveyed had used cannabis in the last 30 days. The highest rate was in Italy (15%) and the lowest in Kosovo (1.4%). More boys reported use in the last 30 days than girls (8.5% versus 5.8%). According to the Cannabis Abuse Screening Test, the prevalence of high-risk cannabis use ranged from 1.4% to 7.3% among all students, with an average of 4.0%. High-risk cannabis use was more common among boys than girls (4.7% versus 3.3%).

In the following sections we briefly summarise recent systematic reviews of evidence on the harms of cannabis use that may be experienced by European cannabis users and that may require health system responses.

## Cannabis use disorders

The 2019 Global Burden of Disease (GBD) study estimated that 0.5% of the EU adults aged 15–64 met criteria for a cannabis use disorder in 2019 (95% CI: 0.2 to 0.9%). This did not differ from the 2010 GBD estimate (0.6%, 95% CI: 0.2 to 0.9%). The prevalence of CUD varied substantially from 0.1% in Turkey to 1.2% in the UK. It was difficult to identify trends in the prevalence of CUD between 2010 and 2019 because there were large confidence intervals around these estimates for most countries [37].

In 2021, there were 97,000 treatment entries for cannabis problems (55,000 for the first time) in the 25 countries that reported to EMCDDA. These entries accounted for almost half (45%) of all persons entering treatment for a drug problem [17]. There were large differences in treatment rates between countries: there were less than 2 treatment entries for cannabis problems per 100,000 adults in Bulgaria and Slovenia in 2019 compared with more than 100 treatment entries per 100,000 adults in Malta [16].

Data were available from 22 countries in the EU in the years 2010 and 2019 (comprising 76% of the population aged 15 to 64 in all 28 EU countries in 2019). The rate of people entering treatment with cannabis as their primary problem increased from 27.0 (95% CI: 17.2 to 36.8) to 35.1 (95% CI: 23.6 to 46.7) per 100,000 adults. Only the Netherlands, Finland and Slovenia showed a significant decline in treatment rate in this period. Treatment rates significantly increased in ten countries: modestly in Greece, Poland, Austria, Slovakia, and Portugal, and more markedly in Romania, Belgium, France, Malta, and Sweden. In the 17 countries with continuous treatment data between 2010 and 2019, treatment rates ceased increasing in 2015 and have plateaued since then. The interpretation of these trends is complicated by the fact that there was a decline in the number of treatment units providing data since 2014.

## The adverse health effects of cannabis

Research on the harms of cannabis use is incomplete and there are also debates about whether all of the associations reported in these studies between regular cannabis use and adverse health outcomes are causal [25, 49]. Most of our knowledge of the adverse effects of cannabis use comes from studies of cannabis users in high income countries (HICs) all of which until very recently prohibited nonmedical cannabis use, namely, Australia, Canada, Germany, the Netherlands, New Zealand, Sweden and the United States of America [25].

Under a policy of prohibition, the adverse health effects of cannabis are modest by comparison with those of alcohol, tobacco and the opioids [25]. This is for at least two reasons: the prevalence of regular cannabis use is much lower in these countries than the regular use of alcohol and tobacco; and the risks of many adverse health effects appear to lower for cannabis than alcohol and tobacco [18, 32, 47].

### Acute harms

Cannabis does not appear to cause fatal overdoses because it does not cause respiratory depression like the opioids or acute cardiovascular events like cocaine [20, 31, 33]. In a minority of cannabis users the acute effects of cannabis can include severe anxiety, heart palpitations, and psychotic symptoms [11]. These events are more likely to occur among naïve users and among persons using cannabis products with high levels of THC [46, 48]. There has been an increase in the prevalence of persons presenting to emergency departments with these adverse effects since cannabis edibles and cannabis extracts became legally more available in Colorado [42] and Canada [44, 45]. The number of children who require hospital treatment after accidentally ingesting edible cannabis products has increased since the sale of cannabis edibles was legalised in Canada and the USA [44, 45, 51].

The acute adverse effect of cannabis use of greatest public health significance is an increased risk of road crashes, if users drive while intoxicated [24, 46]. The risk is smaller than that for alcohol-impaired drivers because drivers who have used cannabis are less impaired and appear to be more aware of their impairment and so less likely to take risks [46, 49].

Women of reproductive age who regularly use cannabis during their pregnancies may reduce their babies’ birth weights [21, 23]. They may also have poorer birth outcomes, such as small for gestational age, preterm delivery, and neonatal intensive care admission, a lower Apgar score at birth, and a smaller infant head circumference [38]. A number of cohort studies suggest that children exposed to cannabis in utero may have a higher risk of cognitive and behavioural problems in childhood, although these studies have not controlled for confounders such as, tobacco and alcohol use, the genetic risk of developing childhood disorders and the effects of adverse childhood experiences [14, 46].

### Regular cannabis use and the risks of cannabis dependence

The daily use of cannabis increases the risk of developing cannabis dependence [50], a disorder in which affected persons find it difficult to control or cease using cannabis even when they recognise that it is harming them [5, 8]. Abrupt cessation of use can produce a cannabis withdrawal syndrome [3]. There is emerging evidence that persons who use cannabis for medical purposes, such as treating chronic pain, may be at increased risk of developing cannabis use disorders ( [27]).

Cannabis dependence is a common reason for seeking addiction treatment in many high income countries [24, 47].This was also true in the Netherlands which has not enforced criminal penalties for cannabis use since the mid-1970s [41].

### Correlates of Cannabis dependence

Cannabis dependence is associated with an increased risk of a number of adverse psychosocial outcomes [46] that include: psychoses and depressive and anxiety disorders; suicidal ideation; the use of other illicit drugs; cognitive impairment; poor educational outcomes; and antisocial behaviour, such as violence. There is a debate about whether cannabis use is a contributory cause of these outcomes or whether the associations are explained by shared risk factors, or reverse causation [24, 25, 46].

Regular cannabis use in young people is associated with poorer cognitive performance [36, 53]. Impairment is most evident when young people use cannabis daily, as a substantial minority do throughout adolescence [53]. Young people who perform poorly in primary school are more likely to become regular cannabis users but it is probable that daily cannabis use adversely affects their educational outcomes by impairing their ability to learn while at school and by increasing the chance that they will leave school early [55]. Cognitive performance improves with abstinence, although there are conflicting findings on whether the recovery is complete [52].

There is reasonably convincing evidence that daily cannabis use that is initiated in early adolescence can bring forward the onset of psychoses in persons with a personal or family history of psychiatric disorder [26, 39, 43]. Continued daily cannabis use also worsens the course of psychotic disorders [2].

It is uncertain whether the association between daily cannabis use and depression and anxiety disorders is causal [54]. The relative risks are modest (RR < 2) and reverse causation (e.g. cannabis is used for self-medication) and shared risk factors (including genetic factors) are plausible alternative explanations that cannot be excluded [28]. It is plausible that cannabis, like alcohol, is initially used to medicate low mood and anxiety and that cannabis use becomes a regular way of dealing with low mood, producing tolerance and withdrawal symptoms. Cannabis dependence may then be added to the depressive or anxiety disorder, worsening its course, and making it difficult for these young people to develop more effective ways of coping with their disordered mood.

### The long-term health effects of regular cannabis use

The effects of using cannabis daily over decades on developing common diseases, such as cancer and heart disease, are poorly understood [24, 46]. This is in part because very few cannabis users have engaged in daily use for this long under cannabis prohibition. It has also been difficult to disentangle the effects of cannabis use from those of tobacco smoking and alcohol use that are more common among regular cannabis users. Estimates of the contribution of cannabis use to the Global Burden of Disease (GBD; [10]) do not include any long-term adverse health effects for this reason. Studies of the contribution of cannabis to disease burden suggest that it has much smaller impacts than alcohol, tobacco, heroin and cocaine in HICs. Its largest impacts are attributed to cannabis dependence and road crashes caused by cannabis impaired drivers [30].

## Health system responses to cannabis use disorders

### Public education, screening and brief intervention for hazardous cannabis use

In countries that prohibit the use of cannabis, it is difficult to advise users on ways to reduce the risks of cannabis use disorders because the advocacy of "safer" ways of using cannabis may criticised for condoning or normalising the use of an illicit drug. Information can still be given on how to reduce the risks of cannabis use e.g. by explaining that the risk of cannabis dependence increases with regular use, especially when use is daily or near daily over periods of weeks or months (e.g., [19]).

It is good clinical practice to screen persons who present for medical treatment in primary care for hazardous cannabis use. The focus would be on patients in whom there is likely to be an increased prevalence of cannabis use and/or elevated risk of harm, such as, young adults with respiratory problems, persons that use illicit drugs, and pregnant patients. These patients could be routinely asked about their cannabis use when inquiring about their use of tobacco, alcohol and other drugs. Young adults presenting with symptoms of anxiety and depression could be screened because these disorders are common among young people with cannabis use problems [22, 35].

The primary aim of screening is to identify persons who are at risk of cannabis-related harm and to intervene to reduce this risk. Cannabis-dependent persons who do not want to stop using cannabis can be given advice on how to minimise the potential adverse health effects of their use [19]. For example, they can reduce the respiratory risks of cannabis smoking by changing their route of administration to swallowing or using a vaporiser. The next best options may be to advise against smoking tobacco and cannabis together, and to discourage the use of waterpipes, deep inhalation, and breath holding. We should also advise cannabis users against driving or operating machinery after using cannabis or feeling its effects. Such advice may help dependent cannabis users to reduce some of the risks of their use.

Brief intervention in primary care or other health settings may be effective in helping persons who wish to reduce or cease using cannabis. Early trials of dependent cannabis users reported that one or two sessions of counselling improved outcomes up to 6 months later, compared to a wait-list control group [9, 56]. More recent systematic reviews of these studies have found weak evidence for their effectiveness [29]. If a brief intervention is ineffective, the person can be referred to specialist treatment.

### Specialist treatment for adult cannabis use disorders

Specialist treatment may be needed for adults with cannabis use disorders who have not been able to cease or moderate their cannabis use with self-help or brief interventions [8]. Persons with cannabis use disorders can be successfully treated on an outpatient basis to reduce cannabis use and the severity of their cannabis-related problems [4, 8]. Treatment outcome rates for Cognitive Behavioural Therapy (CBT) and Motivational Enhancement Therapy (MET) are like those for alcohol use disorder treatment [8]. In studies of combined CBT and MET, short term outcomes (median 120 days) show a 25% reduction in cannabis use and a doubling of abstinence rates, compared to non-active treatment (Gates, et al., 2016). Sustained abstinence nonetheless remains modest in many of these trials e.g., 15% at 6-and 12-months [9, 57]. There is currently limited evidence to support pharmacological approaches for treatment of cannabis use disorders [8].

### Withdrawal management

Nearly half (47%) of persons who seek help for cannabis use disorders report withdrawal symptoms [8]. These symptoms may be a barrier to achieving abstinence, and may need to be managed with psychological and supportive strategies and possibly pharmacotherapy. There is currently insufficient evidence to support any pharmacological approaches for cannabis withdrawal, but some medications are being used ‘off label’ to reduce severity of withdrawal. It is unlikely cannabis withdrawal requires an inpatient admission, except in the case of severe comorbid psychiatric or medical conditions [7].

### Managing comorbid substance use and mental disorders

The most common types of comorbidity among persons with cannabis use disorders are other substance use disorders, such as, alcohol, sedative, and opiate disorders [12, 58]. Treatment programs for cannabis dependence that exclude persons with other substance use disorders exclude an important part of the target population. Treatment programs for cannabis use disorders also need to address alcohol and other drug use disorders among patients affected by them.

In surveys, persons with cannabis use disorders report higher rates of anxiety and affective disorders than persons who do not have this diagnosis and these disorders increase treatment-seeking [13]. In treating cannabis dependence, treatment services may need to improve their recognition and treatment of comorbid anxiety and affective disorders by using brief, valid and reliable screening tests for these symptoms [8].

Specialist mental health services often struggle to respond to cannabis use disorders in young adults with schizophrenia, among whom up to a third may use cannabis daily [6, 26]. The treatment of schizophrenic patients is complicated by the presence of cognitive deficits, poor motivation and compliance, impaired social functioning, limited support networks, and the use of neuroleptic medication [6, 26]. Comprehensive programs that integrate psychiatric and substance use interventions are ideal, but cost is a barrier to their widespread use.

### Adolescent cannabis dependence

Many young persons who use cannabis may not be motivated to change because they do not see any need to change, despite the concerns of parents and difficulties at school. There is less available high-quality evidence for adolescent cannabis dependence treatment, compared to research on adult interventions. The strongest support is for behaviourally based family-systems based therapy, individual CBT and MET. There may be additional benefit in combining these approaches with Contingency Management (CM) where treatment attendance and positive cannabis use outcomes are incentivised via money or vouchers (Connor, et. al., 2021). Promising research using motivational approaches (e.g., [40]), which include the "Check-up" approach to enhance motivation with this group, suggest that further investment in early intervention efforts is warranted.

## Conclusions

Cannabis use disorders are among the most common forms of drug problem treated in many countries in Europe. Public education about the risks of cannabis use may be one way of reducing the prevalence of the more common, less severe forms of cannabis use disorders. Good advice on self-help strategies for quitting or cutting down may reduce the need for professional assistance in some of these cases. Individuals whose problems do not respond to self-help, may need treatment on an outpatient basis. Special issues that may need to be addressed in treatment include comorbidity between cannabis and other types of drug dependence, and between cannabis use disorders and other mental disorders. The special needs of adolescents with cannabis problems or manifest disorders may require careful attention and an enhanced prevention focus.
